# Population Genetic Structure and Demographic History of *Atrina pectinata* Based on Mitochondrial DNA and Microsatellite Markers

**DOI:** 10.1371/journal.pone.0095436

**Published:** 2014-05-01

**Authors:** Dong-Xiu Xue, Hai-Yan Wang, Tao Zhang, Jin-Xian Liu

**Affiliations:** 1 Key Laboratory of Marine Ecology and Environmental Sciences, Institute of Oceanology, Chinese Academy of Sciences, Qingdao, Shandong, China; 2 Department of Marine Organism Taxonomy and Phylogeny, Institute of Oceanology, Chinese Academy of Sciences, Qingdao, Shandong, China; 3 University of Chinese Academy of Sciences, Beijing, China; School of Environment & Life Sciences, University of Salford, United Kingdom

## Abstract

The pen shell, *Atrina pectinata*, is one of the commercial bivalves in East Asia and thought to be recently affected by anthropogenic pressure (habitat destruction and/or fishing pressure). Information on its population genetic structure is crucial for the conservation of *A. pectinata*. Considering its long pelagic larval duration and iteroparity with high fecundity, the genetic structure for *A. pectinata* could be expected to be weak at a fine scale. However, the unusual oceanography in the coasts of China and Korea suggests potential for restricted dispersal of pelagic larvae and geographical differentiation. In addition, environmental changes associated with Pleistocene sea level fluctuations on the East China Sea continental shelf may also have strongly influenced historical population demography and genetic diversity of marine organisms. Here, partial sequences of the mitochondrial Cytochrome c oxidase subunit I (COI) gene and seven microsatellite loci were used to estimate population genetic structure and demographic history of seven samples from Northern China coast and one sample from North Korea coast. Despite high levels of genetic diversity within samples, there was no genetic differentiation among samples from Northern China coast and low but significant genetic differentiation between some of the Chinese samples and the North Korean sample. A late Pleistocene population expansion, probably after the Last Glacial Maximum, was also demonstrated for *A. pectinata* samples. No recent genetic bottleneck was detected in any of the eight samples. We concluded that both historical recolonization (through population range expansion and demographic expansion in the late Pleistocene) and current gene flow (through larval dispersal) were responsible for the weak level of genetic structure detected in *A. pectinata*.

## Introduction

Population connectivity plays a significant role on both evolutionary and ecological time-scales in both terrestrial and marine species [1]. Dispersal and gene flow mainly occur via dispersion of larvae, gametes, and asexual propagules in sedentary or sessile marine organisms, which are likely to bear principal responsibility for population connectivity [2]. Marine species are usually expected to show lower geographical differentiation than terrestrial species, as a result of some or a combination of the following: lack of geographical barriers, large population sizes, high fecundity, wide range of distribution, and long pelagic larval phase [3]–[5]. Indeed, some marine bivalves lack geographical differentiation over large geographic scales [6], [7]. However, genetic differentiation over either large or fine geographical scales has been reported in many marine bivalves, including clams [4], [8], oyster [9], [10], scallop [11], [12] and mussels [13]. These results suggest that dispersal of pelagic larvae may be limited by ecological or hydrographical barriers, such as temperature, salinity, or currents.

In the coasts of China and Korea, the complex hydrology and bathymetry of Bohai Sea and Yellow Sea, including numerous bays, islands, and gyres, suggest the potential for restricted dispersal of pelagic larvae and geographical differentiation of marine bivalve in this region. Indeed, previous studies on *Scapharca broughtonii* [14], *Mactra veneriformis* [15], *Chlamys farreri* [12] and *Mactra chinensis* [8] reveal significant geographical differentiation and population structure in this region.

Moreover, Pleistocene glaciations have played an important role in shaping patterns of genetic diversity [16]. The Bohai Sea, Yellow Sea, and East China Sea Shelf were exposed during the Pleistocene ice ages [17]. For example, during the last glacial maximum (LGM), the sea level was 130–150 m below present sea level [18]. Within some 8000 years of the last deglaciation, the coastline migrated about 1200 km landwards from the western border of the Okinawa Trough to the western coast of the modern Bohai Gulf, resulting in the flooding of the East China Sea Shelf [17], [19]. This sea level rise resulted in an expansion in range for marine species that inhabit the East China Sea Shelf [19]. For marine organisms inhabiting the East China Sea, historical population demographic expansion might occur with the range expansion following the LGM, which might result in: (i) a star-like phylogeny with few high-frequency ancestral haplotypes and numerous low-frequency haplotypes separated from the ancestral ones by a few mutational steps, (ii) low levels of genetic subdivision among populations, and (iii) a Poisson distribution of pairwise nucleotide differences among haplotypes indicating a sudden increase in effective population size [20].

The pen shell, *Atrina pectinata*, is common along the coasts of China, North Korea, South Korea and Japan. The fishery operates primarily along the coasts of Bohai Sea and Yellow Sea in China and along the eastern coast of Yellow Sea in North Korea [21]. It is a long lived (about 7 years) and sedentary bivalve found in habitats ranging from muddy to sandy sediment, and from subtidal zone to a depth of 100 m [22], [23]. *A. pectinata* is a broadcast spawner with external fertilization, and spawning times vary among populations, triggered by environmental cues such as temperature [22]. In Bohai Sea and Yellow Sea, spawning generally occurs between June and August [22]. Although sedentary as adult, *A. pectinata* has a pelagic larval phase that lasts about 30 days [24] and mean fecundities of 29 million eggs per year [25], which suggests high dispersal potential.

*A. pectinata* is known to exhibit extensive morphological variations and five morphological forms of *A. pectinata* exist along the coast of China [26]. By using mitochondrial DNA (COI) and nuclear DNA (ITS) analysis, study of Liu et al. [26] support the existence of five cryptic species within *A. pectinata,* which generally correspond to the five morphological forms observed. Four of the five cryptic species mainly distribute in the South China Sea. *A. pectinata* populations distributed in the Bohai Sea, Yellow Sea, and East China Sea correspond to a single cryptic species (Lineage I) found by Liu et al. [26].

As a lucrative commercial fishery, *A. pectinata* has experienced severe population declines due to over-fishing, habitat loss, pollution, and other factors in the past decades [21], [27]. Knowledge of population genetic structure is essential for sustainable management, conservation, and rehabilitation of species. However, with little research conducted to date, information on population genetic structure of *A. pectinata* along the coasts of Bohai Sea and Yellow Sea is still limited.

An *et al.* [21] studied three populations of *A. pectinata* along the west coast of South Korea using 13 microsatellite loci, and no significant genetic differentiation are detected. Considering its long pelagic larval phase and iteroparity with high fecundity, the genetic structure of *A. pectinata* could be weak at a fine scale. However, the unusual oceanographic characters around Bohai Sea and Yellow Sea, which may act as hydrographical and ecological barriers to larval dispersal, is likely to result in genetic differentiation among populations in this species.

In the present study, samples of *A. pectinata* were collected from eight geographic locations along coast of China and North Korea. Microsatellite markers and a portion of the mitochondrial COI gene were employed to address three key issues. First, we have estimated population genetic structure among populations. In particular, we attempted to test whether populations associated with the bays and gyres around Bohai Sea and Yellow Sea are genetically distinct, as previous studies suggested. Second, we have estimated the historical population demography of *A. pectinata* and discussed its response to climatic fluctuations during the Pleistocene, especially the LGM. Finally, we have tested whether the recent decrease in census population numbers has been accompanied by a genetic bottleneck in any population studied since such bottlenecks can compromise the future adaptive potential of populations.

## Materials and Methods

### Ethics Statement

Ethical approval was not required for this study because no endangered animals were involved. However, all handling of *A. pectinata* specimens was conducted in strict accordance with Animal Care Quality Assurance in China.

### Sample Collection and DNA Extraction

**Figure 1:**
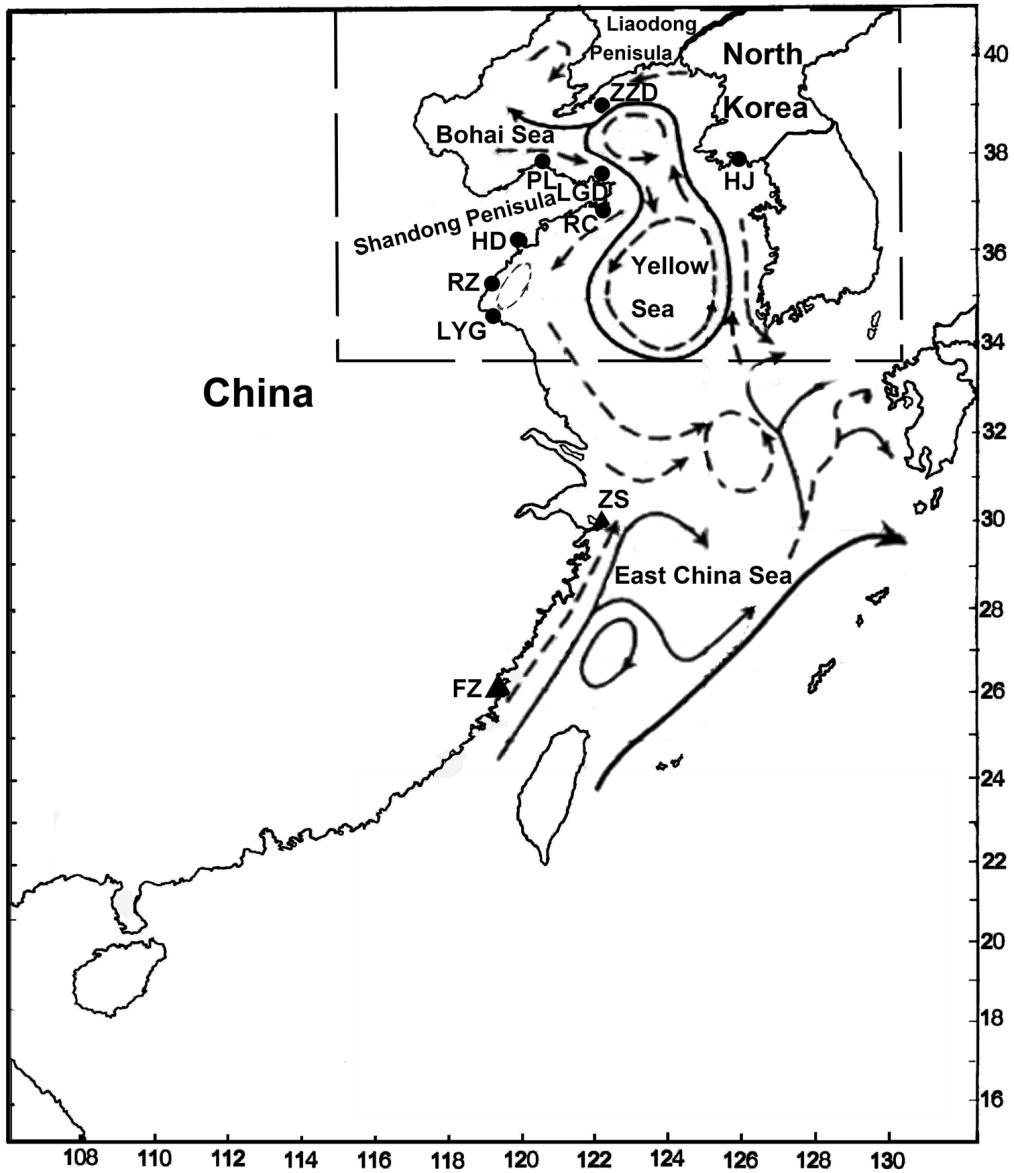
Map of the studied area depicting sample locations and schematic map of mean currents. Populations are marked by abbreviations that correspond to Table 1.

Samples of *A. pectinata* (n = 243) were collected from seven locations in China, and one in North Korea from Bohai Sea and Yellow Sea during 2008–2013 (Table 1, Figure 1). The posterior adductor muscles were preserved in 95% ethanol. Total genomic DNA was extracted from ethanol-fixed adductor muscle tissue using the TIANamp marine animals DNA kit (Tiangen Bio., Beijing, China).

**Table 1:** Sampling information and molecular diversity indices for COI data.

Locality	Abb.	Date of collection	*N*_NU_	COI		
				*N*_MT_(nh, np)	h	π
Zhangzidao, Liaoning	ZZD	11/2008	23	20(12,4)	0.874	0.003
Penglai. Shandong	PL	11/2008	35	20(9,5)	0.653	0.002
Liugongdao, Shandong	LGD	11/2008	32	21(9,4)	0.729	0.003
Rongcheng, Shandong	RC	05/2009	28	20(11,3)	0.805	0.004
Hongdao, Shandong	HD	05/2009	23	20(16,5)	0.947	0.004
Rizhao, Shandong	RZ	06/2009	37	21(13,5)	0.933	0.005
Lianyungang, Jiangsu	LYG	11/2008	30	20(11,6)	0.805	0.003
Haeju, North Korea	HJ	06/2013	35	24(12,5)	0.935	0.006
Zhoushan, Zhejiang*	ZS	–	20	20(14,9)	0.916	0.005
Fuzhou, Fujian*	FZ	–	25	25(17,11)	0.903	0.003

Abbreviation of populations (Abb.), date of collection, number of specimens for nuclear microsatellite analysis (*N*_NU_), and number of specimens for mitochondrial COI analysis (*N*_MT_), number of haplotypes (nh), private haplotypes (np), haplotype diversity (h) and nucleotide diversities (π) based on the COI data for are shown.

*Sequences of Zhoushan and Fuzhou were retrieved from GenBank.

### Mitochondrial COI Sequencing

A portion of the mitochondrial COI gene was amplified for 20–24 individuals per site (Table 1). The universal COI primers LCO1490 and HCO2198 [28] were used for the amplification. PCR reactions and sequencing with both forward and reverse primers were performed followed the protocol described in [29].

### Nuclear Microsatellite Genotyping

All samples of the eight locations were genotyped at eight microsatellite loci originally described by An *et al.* [23]. Eight microsatellite loci, KAP2, KAP9, KAP11, KAP12, KAP20, KAP32, KAP38, and KAP39, were amplified following the PCR protocol described in [30]. Electrophoresis was carried out in the Life Technologies Biotechnology Co., China.

### Data Analysis

The COI sequences were initially aligned using CLUSTAL X2 [31]. Molecular diversity indices such as haplotype diversity (*h*), and nucleotide diversity (*π*) of the COI sequences were calculated in DnaSP 5.10 [32]. Genealogical relationships among haplotypes were further assessed using a minimum spanning tree constructed by Arlequin 3.5 [33]. Microsatellite alleles were scored using GENEMARKER software version 2.2.0 (SoftGenetics, State College, PA, USA). The number of unique alleles (U), observed (*H_O_*) and expected (*H_E_*) heterozygosity were calculated by using the Excel Microsatellite Toolkit [34]. Hardy-Weinberg equilibrium (HWE) and genotypic linkage disequilibrium (LD) were performed by GENEPOP 4.0 [35]. Allelic richness (*A_R_*), inbreeding coefficient (*F_IS_*) were calculated with FSTAT2.9 [36]. The software MICRO-CHECKER 2.2.0 [37] was used to test for technical artefacts such as null alleles, stuttering and large allele dropout. To investigate genetic differentiation among populations, analysis of molecular variance (AMOVA) was performed for both the nuclear microsatellite and mitochondrial COI data using Arlequin 3.5 [33] with 10,000 permutations. Pairwise genetic divergence values between populations were estimated using *F*_ST_ values for microsatellite data and *Φ*_ST_ values for COI sequences with Arlequin 3.5 [33], and significance was adjusted using a Benjamini–Yekutieli correction based on the false discovery rate approach [38]. *D*_A_ distance [39] were calculated using POPTREE2 [40] for microsatellite data. Population pairwise *F*_ST_ values and *D*_A_ distance for microsatellite data were displayed in two dimensions via multidimensional scaling analysis using the SPSS16.0 software. To identify population structure, the software STRUCTURE 2.3 [41], [42] was used to identify clusters of genetically similar populations using a Bayesian approach for the nuclear microsatellite. Ten replicates were run for all possible values of the maximum number of clusters (K) up to K = 9, and for each run, 1,000,000 iterations were carried out after a burn-in period of 100,000 iterations. To detect the number of genetically homogeneous groups (K) that best fit the data, we used Structure Harvester website [43], which implements the Evanno method [41]. Assignment test was also used to clarify the geographical differentiation. Assignment methods have proven to be useful tools in detecting the influence of marine currents on population genetic structure [12], [44]. The likelihood of an individual originating from a given population was estimated by using a Bayesian-based method implemented in the program GeneClass2 [45]. To obtain a conservative estimate of recent migration, an individual was excluded from its sampling site when the probability of exclusion was greater than 99% (*P* or α <0.01). Potential source locality of the excluded individuals were identified based on probabilities larger than 0.1 [46].

Isolation-by-distance (IBD) analyses were conducted for both COI and microsatellite data. Shoreline distances between sampled populations were estimated in km using Google Earth version 4.3 and plotted against genetic distance, pairwise *Φ*_ST_/(1–*Φ*_ST_) and *F*_ST_/(1–*F*_ST_) for COI and microsatellites, respectively [47]. IBD regression analysis was performed online using the IBD web service [48] with 10,000 randomizations of the data. Inferences on historical demographic history were obtained by neutrality tests, mismatch distribution, and Bayesian Skyline Plot based on COI data. As for neutrality test, Tajima’s *D* test [49] and Fu’s *F*s test [50] were calculated using Arlequin 3.5 [33] with 10,000 permutations. Mismatch distribution was constructed for each geographic population to test a model of exponential population growth [51]. A goodness of fit test was performed to test the validity of the sudden expansion model, using a parametric bootstrap approach based on the sum of square deviations (SSD) between the observed and expected mismatch distributions. The raggedness index which measures the smoothness of the mismatch distribution was calculated for each distribution. The demographic expansion parameter (τ), were calculated with Arlequin 3.5 [33]. Changes in effective population size across time were also inferred using Bayesian Skyline method [52] implemented in the program BEAST1.7.5 [53] with 20 groups. Chains were run for 100 million steps that yielded effective sample sizes (ESS) of at least 200 and first 10% was discarded as “burn-in” under the TN93 substitution model from Modeltest 3.7 [54], a strict molecular clock and a stepwise skyline model. All operators were optimized automatically. Results of the analyses were visualized using Tracer 1.5 [55].

To investigate whether any of the populations experienced recent genetic bottlenecks, Wilcoxon sign-rank test for heterozygosity excess was applied under three different models, namely, infinite alleles model (IAM), two-phase model (TPM) and stepwise mutation model (SMM), using the program Bottleneck 1.2.02 [56]. Furthermore, the qualitative test of model shift was performed to calcuate the allele frequency distribution using Bottleneck 1.2.02 [56].

## Results

### Genetic Diversity

**Figure 2:**
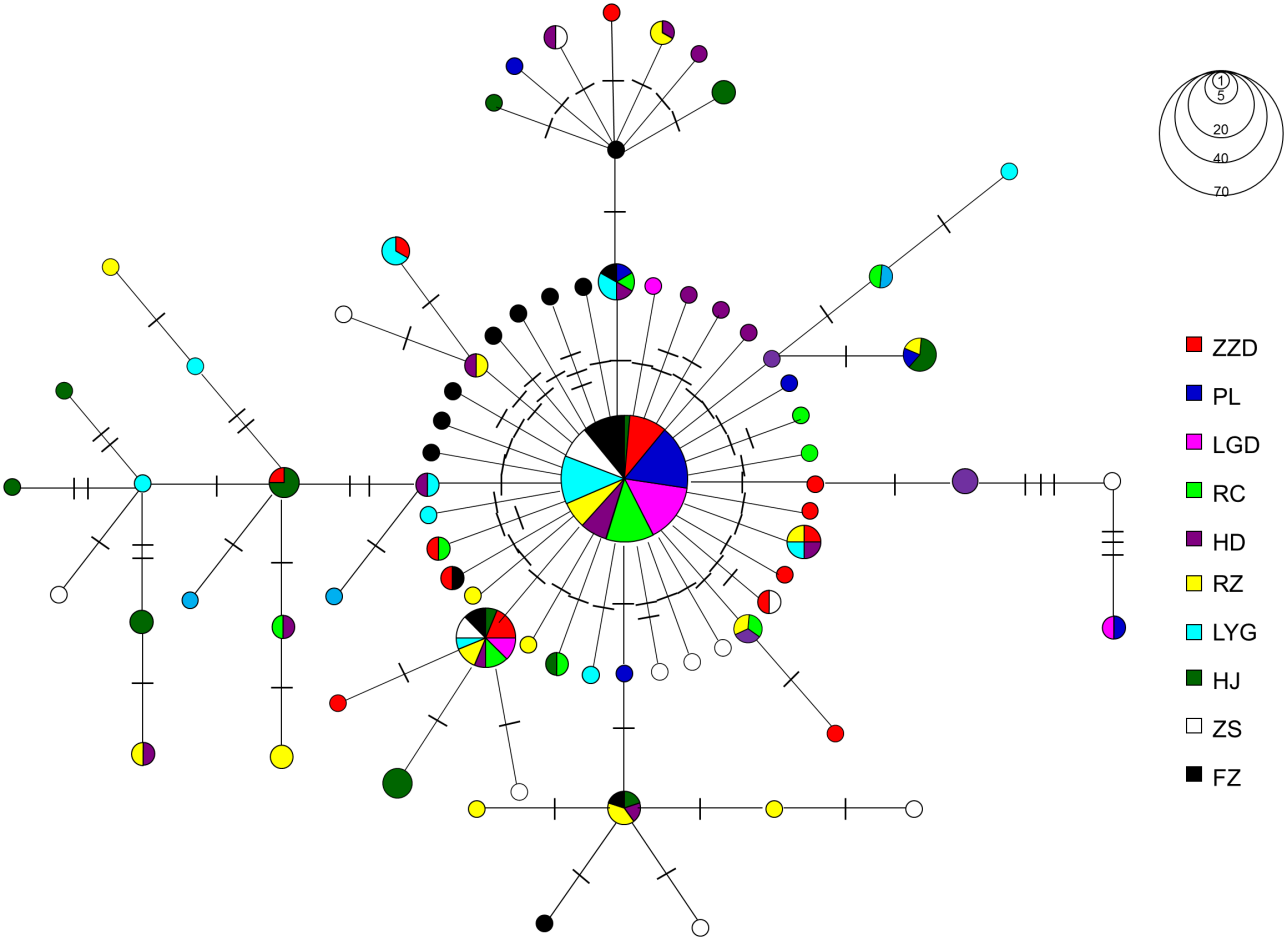
Minimum spanning tree showing genetic relationship among COI haplotypes in *A. pectinata*. Circles represent haplotypes with sizes proportional to their respective frequencies. The population origins of haplotypes are indicated by colors. Tick marks represent deduced numbers of nucleotide substitutions along each branch.

A 625-bp fragment of the COI gene was sequenced for 166 *A. pectinata* adults. An additional 45 sequences of two populations (ZS and FZ) in Southern China coasts were also retrieved from GenBank and incorporated in the analyses [26]. A total of 70 substitutions, all of which is transitions, at 70 polymorphic sites defined 79 haplotypes (Table 1). Haplotype diversity (*h*) ranged from 0.653 to 0.947, and nucleotide diversity (*π*) ranged from 0.002 to 0.006 (Table 1). Haplotype diversity (*h*) and nucleotide diversity (π) of the PL population in Bohai Sea were generally lower than that of other populations. A total of 57 private haplotypes were identified, of which 32 were found in the seven populations in Northern China, five in HJ population in North Korea, and 20 in the two populations from Southern China coast (Table S1). A dominant haplotype (34.6%) was shared by all populations, which was also the dominant one within each population except the HJ population. The haplotype with the second highest frequency (7.6%) was also found in nine populations (except PL population). Fifty-three haplotypes were represented by only one individual. The minimum spanning tree of haplotypes showed no apparent geographical clustering of haplotypes, and a star-shaped genealogy was observed, which is often associated with demographic expansion (Figure 2). The minimum spanning tree confirmed that samples analyzed in the present study corresponded to a single cryptic species (Lineage I) detected by Liu et al. [26].

Summary statistics for eight microsatellite loci in the eight populations from China and North Korea coast are showed in Table 2. A total of 236 alleles were detected across all eight loci, with 18 alleles at the KAP9 and KAP20 loci to 41 alleles at locus KAP32. The parameter allelic richness (*A_R_*), which is independent of sample size, was employed to compare among populations. The total allelic richness (*A_R_*) per locus ranged from 9.70 (KAP20) to 19.52 (KAP38). Average *A_R_* value was highest in LYG (15.86) and lowest in HJ (12.34). A total of 39 alleles were unique to a single population, ranging from two in LGD to eight in RZ. The observed and expected heterozygosities per locus varied from 0.679 (KAP20) to 0.930 (KAP11), and from 0.742 (KAP39) to 0.941 (KAP11), respectively. There was no significant difference in the average allelic richness, observed and expected heterozygosity among populations (Kruskal-Wallis test, *d*f = 7, *P*>0.05). No linkage disequilibrium was detected for 224 combinations in the 8 populations. Eight cases of locus-population combination out of 64 showed significant departure from Hardy-Weinberg equilibrium (HWE) after sequential Bonferroni correction (initial *P*<0.002). Departures were primarily related to excess of homozygosity at locus KAP32. No population deviated significantly from HWE by global tests across all loci. Analysis using MICRO-CHECKER suggested presence of null alleles at locus KAP32 in all populations. Thus, KAP32 was eliminated from subsequent analyses.

**Table 2:** Summary of the statistics for eight microsatellite loci.

Locus			ZZD	PL	LGD	RC	HD	RZ	LYG		HJ		Total
N			23	35	32	28	23	37	30		35		243
KAP2-FAM	*A_R_(U)*		17.00(1)	17.47(0)	15.58(0)	17.54(0)	18.00(2)	13.79(1)	16.60(0)		11.18(1)		15.98
	*H_O_*		0.957	0.914	1.000	0.893	0.957	0.973	0.867		**0.686**		0.901
	*H_E_*		0.932	0.937	0.926	0.910	0.940	0.910	0.931		0.899		0.925
	*Fis*		−0.027	0.025	−0.081	0.019	−0.018	−0.07	0.070		0.301		0.019
KAP9 -HEX	*A_R_(U)*		9.00(0)	10.58(1)	8.72(0)	9.00(0)	8.95(0)	8.25(1)	9.59(0)		10.30(0)		10.25
	*H_O_*		0.783	0.829	0.656	**0.643**	0.826	0.649	0.667		0.886		0.741
	*H_E_*		0.822	0.801	0.787	0.820	0.825	0.777	0.807		0.841		0.813
	*Fis*		0.049	−0.035	0.168	0.219	−0.001	0.167	0.177		−0.047		0.017
KAP11 -FAM	*A_R_(U)*		18.00(0)	17.82(0)	16.28(0)	16.83(1)	17.00(0)	16.81(0)	17.77(1)		15.19(0)		17.87
	*H_O_*		0.870	0.943	0.938	0.893	0.957	0.946	0.867		1.000		0.930
	*H_E_*		0.947	0.941	0.937	0.936	0.932	0.931	0.935		0.926		0.941
	*Fis*		0.083	−0.003	−0.001	0.047	−0.027	−0.017	0.074		−0.071		0.017
KAP12 -FAM	*A_R_(U)*		17.60(0)	15.88(0)	19.04(0)	16.06(0)	15.56(0)	16.24(1)	19.95(2)		15.08(1)		18.95
	*H_O_*		0.826	0.857	0.938	0.893	0.957	0.838	0.933		**0.943**		0.897
	*H_E_*		0.900	0.886	0.930	0.886	0.848	0.872	0.916		0.923		0.904
	*Fis*		0.084	0.033	−0.008	−0.008	−0.131	0.039	−0.020		0.032		0.010
KAP20 -TAMRA	*A_R_(U)*		9.91(0)	8.74(2)	7.54(0)	9.26(0)	9.83(1)	10.59(2)	9.53(0)		9.00(0)		9.70
	*H_O_*		0.652	0.600	0.625	0.643	0.739	0.703	0.633		0.829		0.679
	*H_E_*		0.794	0.621	0.715	0.814	0.787	0.820	0.711		0.812		0.815
	*Fis*		0.182	0.035	0.127	0.213	0.063	0.144	0.111		0.050		0.125
KAP32 -FAM	*A_R_(U)*		19.52(1)	18.77(1)	20.63(1)	18.34(2)	20.00(3)	16.03(0)	20.21(1)		14.00(0)		18.44
	*H_O_*		**0.739**	**0.714**	0.750	**0.607**	0.682	**0.676**	**0.621**		0.914		0.719
	*H_E_*		0.941	0.925	0.951	0.925	0.949	0.930	0.931		0.921		0.939
	*Fis*		0.218	0.23	0.214	0.348	0.287	0.276	0.337		0.007		0.272
KAP38 -HEX	*A_R_(U)*		19.65(1)	17.26(1)	18.40(0)	19.93(0)	18.78(0)	18.08(2)	17.44(1)		15.00(1)		19.52
	*H_O_*		0.913	0.857	0.813	0.821	1.000	0.892	0.833		0.800		0.860
	*H_E_*		0.926	0.922	0.882	0.940	0.934	0.899	0.914		0.901		0.918
	*Fis*		0.014	0.071	0.08	0.128	−0.072	0.008	0.090		0.066		0.054
KAP39 -HEX	*A_R_(U)*		14.65(1)	9.95(1)	12.16(1)	11.91(0)	14.65(2)	9.91(1)	14.11(1)		10.12(0)		12.84
	*H_O_*		0.739	0.600	0.781	0.679	0.783	**0.568**	0.800		0.914		0.728
	*H_E_*		0.762	0.596	0.783	0.778	0.757	0.706	0.779		0.770		0.742
	*F_is_*		0.031	−0.007	0.002	0.13	−0.034	0.199	−0.028		−0.101		0.039
		**Average**											
	*A_R_*		15.82	14.69	14.93	15.05	15.50	13.88	15.86	12.34		18.44	
	*H_O_*		0.820	0.800	0.821	0.781	0.888	0.795	0.800	0.857		0.820	
	*H_E_*		0.869	0.815	0.851	0.869	0.861	0.845	0.856	0.872		0.865	

Sample size (N), allelic richness (*A_R_*), number of unique alleles (U), observed heterozygosity (*H_O_*), expected heterozygosity (*H_E_*) and inbreeding coefficient (*F_IS_*). Bold type indicates significant deviations from Hardy-Weinberg equilibrium after Bonferroni correction (*P*<0.0018).

### Population Genetic Structure

**Figure 3:**
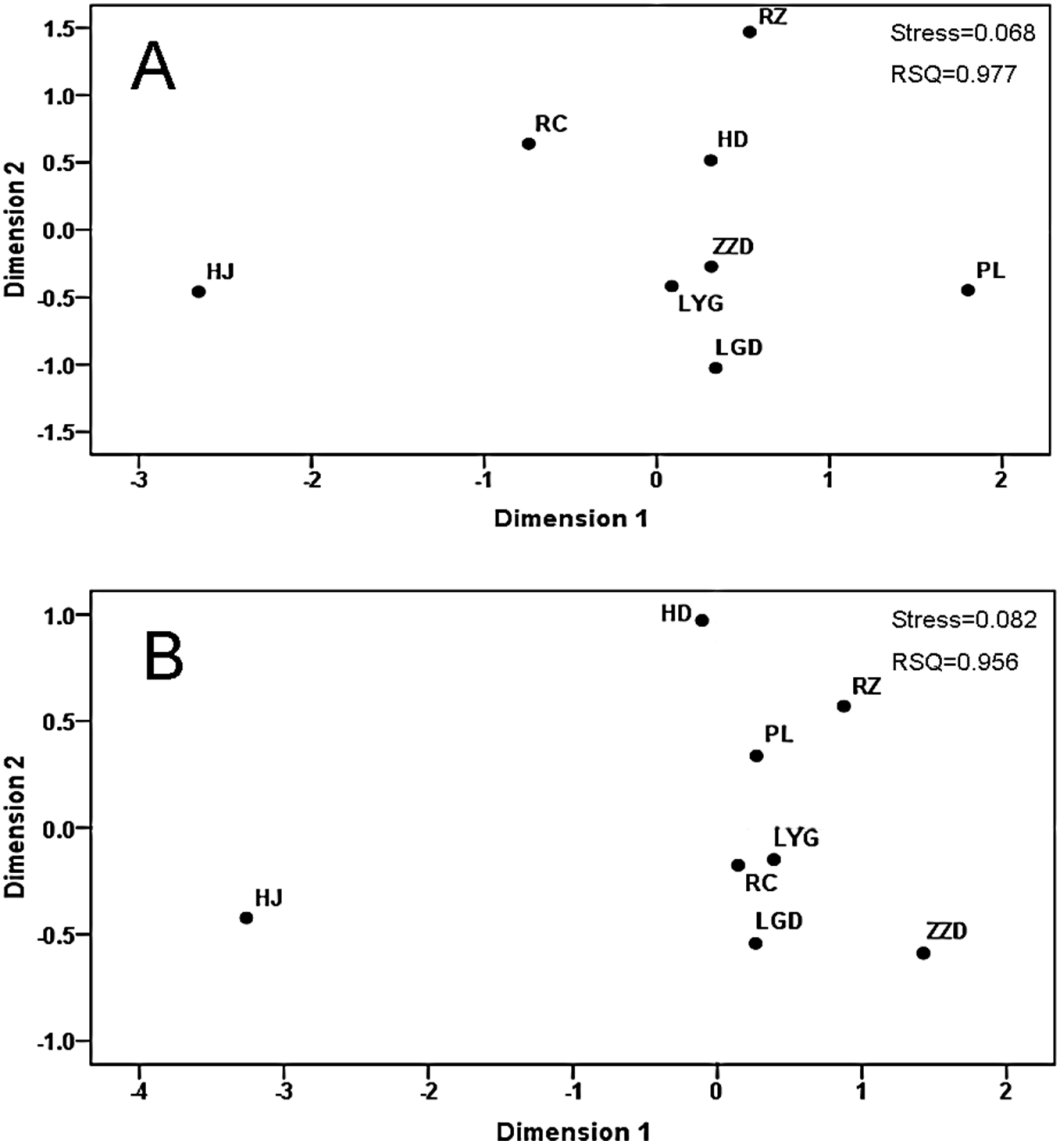
Multidimensional scaling plots based on *F_ST_* values (A) and *D_A_* distance (B) between *A. pectinata* populations.

AMOVA analyses were conducted for COI datasets with two groups: the Northern group (eight populations in coast of Northern China and North Korea) and the Southern group (ZS and FZ population). For microsatellite datasets, the AMOVA analyses were also conducted with two groups: the Northern China group (seven populations) and the North Korea group (HJ population). The AMOVA indicated no significant genetic differentiation at all levels (among groups, among populations within groups, and within populations) for COI datasets, while results for the nuclear microsatellite datasets (Table 3) indicated shallow but significant amount of variance at two levels (among populations within groups and within populations). Pair-wise *Φ*_ST_ values based on COI data were low (–0.022∼0.081), and only two (both involving the HJ samples) out of 45 pair-wise *Φ*_ST_ values were significant after a Benjamini–Yekutieli correction, indicating high population connectivity (Table S2). Pairwise *F*_ST_ values based on nuclear microsatellite data ranged from –0.0026 (between LYG and ZZD) to 0.0233 (between PL and HJ) (Table 4). All of the seven samples in China were significantly different from HJ population (*P*<0.05), and five of them were significant after a Benjamini–Yekutieli correction (*P*<0.01273). Based on microsatellite datasets, the lowest *D*_A_ value was between PL and LYG (0.1390) and the highest *D*_A_ value was between ZZD and HJ (0.2489) (Table 5). Both the multidimensional scaling plots based on *F*_ST_ values and *D*_A_ values for microsatellite data showed that HJ population was separated from all the other populations (Figure 3). The Structure analysis showed that the number of genetic groups (K value) best fitting our data was K = 2 (Figure S1). The eight samples did not show any distinct structure to suggest that they are individual populations. The analysis revealed the presence of two sub-clusters in eight populations, indicating that there is a mix of two clusters within all of the eight samples (Figure S1). An exclusion test conducted in GeneClass analysis showed that 239 (98.35%, the remaining six individual from HJ) can be assigned to two or more of the other sampling locations, and for 204 (85.36%) of them, the potential source population with the highest probability was the sampling location. There was no evidence of isolation by distance for both microsatellite and COI datasets (*P*>0.05).

**Table 3:** Analysis of molecular variance (AMOVA) based on microsatellite datasets.

Source of variation	df	Variance components	Percentage of variation	*F-*statistics
Among groups	1	0.034	1.13	F_CT_ = 0.0113
Among populations within groups	6	0.013	0.42	F_SC_ = 0.0042*
Within populations	478	2.986	98.45	F_ST_ = 0.0155**
Total	485	3.033		

*Significant at *P*<0.05.

**significant at *P*<0.01.

**Table 4:** Matrix of pairwise *F_ST_* values (below diagonal) and Nei’s genetic distance (*D_A_*) values (above diagonal) between populations based on microsatellite datasets.

	ZZD	PL	LGD	RC	HD	RZ	LYG	HJ
ZZD	-	0.1791	0.1660	0.1639	0.1921	0.1646	0.1470	0.2489
PL	0.0060	-	0.1447	0.1585	0.1633	0.1427	0.1390	0.2236
LGD	0.0003	0.0050	-	0.1728	0.1716	0.1731	0.1490	0.2215
RC	0.0009	**0.0135**	0.0058	-	0.1718	0.1524	0.1541	0.2174
HD	0.0035	0.0030	0.0056	0.0011	-	0.1515	0.1634	0.2192
RZ	0.0062	**0.0092**	**0.0103**	0.0030	−0.0003	-	0.1596	0.2389
LYG	−0.0026	0.0035	0.0015	−0.0012	−0.0010	0.0070	-	0.2235
HJ	0.0143	**0.0225**	**0.0142**	0.0090	**0.0136**	**0.0183**	**0.0119**	-

Significant values after a Benjamini–Yekutieli correction based on the false discovery rate approach (*P*<0.0127) are highlighted in bold.

**Table 5:** Tajima’s *D*, Fu’s *F*s and parameters of the mismatch distribution for populations based on COI data.

Population	Tajima’s D	Fu’s Fs	τ	SSD(P_SSD_)	Raggedness(P_r_)
ZZD	−2.104**	−7.022**	1.387	0.002(0.847)	0.030(0.863)
PL	−2.225**	−4.085**	2.121	0.020(0.489)	0.082(0.672)
LGD	−1.786*	−2.121	5.470	0.012(0.771)	0.030(0.946)
RC	−1,972**	−4.691**	0.398	0.276(0.007**)	0.034(1.000)
HD	−1.929*	−12.506**	2.766	0.004(0.588)	0.039(0.590)
RZ	−1.575*	−5.568**	2.000	0.003(0.757)	0.021(0.817)
LYG	−1.517	−6.226**	0.639	0.011(0.585)	0.061(0.574)
HJ	−0.587	−2.713	4.096	0.034(0.018*)	0.092(0.012*)
**Northern**	−2.229**	−26.523**	1.644	0.001(0.805)	0.013(0.918)
ZS	−2.078**	−8.200**	1.125	0.130(0.022*)	0.023(0.986)
FZ	−2.256**	−15.501**	2.240	0.002(0.771)	0.048(0.505)
**Southern**	−2.380**	−26.655**	1.593	0.001(0.747)	0.030(0.893)
**Total**	−2.331**	−26.444**	1.568	0.001(0.867)	0.014(0.899)

Time since the population expansion (τ), Sum of square deviations (SSD) and its p-value (P_SSD_) for test of the validity of the sudden expansion model, Harpending’s Raggedness index (Raggedness) and its p-value (P_r_) for the test of goodness-of-fit.

*Significant at *P*<0.05;

**significant at *P*<0.01.

### Population Demography

**Figure 4:**
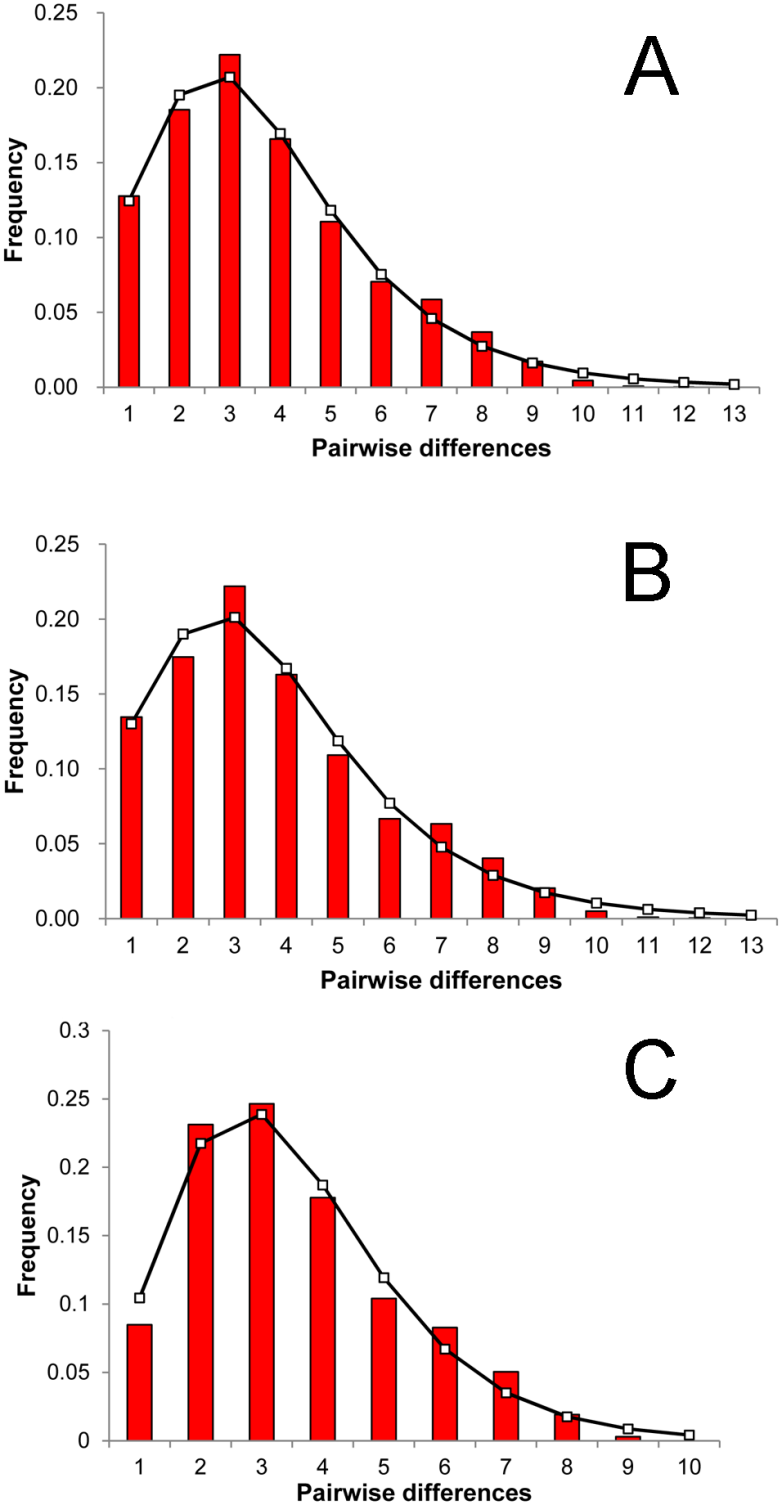
Observed pairwise differences and expected mismatch distribution. Observed pairwise differences (bars) and expected mismatch distribution (solid line) under the sudden expansion model for (A) the global group, (B) northern group, and (C) southern group calculated for COI sequences.

**Figure 5:**
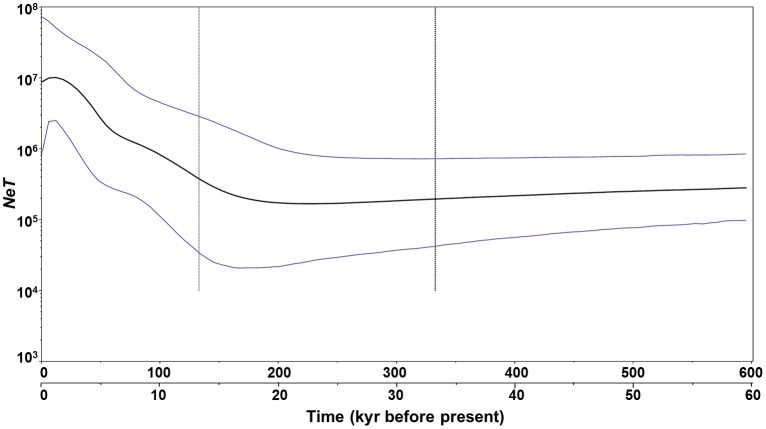
Demographic history of *A. pectinata* estimated using Bayesian skyline plots from COI sequences. The figure shows changes of *NeT* (*Ne* = effective population size; *T* = generation time) through time. Shown at the bottom of the figure on a separate axis are times under an alternative (10×faster) calibration rate for COI gene. Black lines represent median estimates of *NeT*; light lines are the upper and lower 95% highest posterior density (HPD) limits of *NeT*; and the vertical light dashed line represents the median estimates of time to most recent common ancestor (TMRCA). The upper 95% HPD on TMRCA is at the right of the plot, whereas the lower 95% HPD is the black dashed line to the left of median. The y-axis is plotted on a logarithmic scale.

Results of Tajima’s *D* and Fu’s *F*s tests for the 10 populations, the northern group, the southern group, and the global group (all populations combined) are shown in Table 5. Values of both tests were significant and negative for all of the three groups, suggesting a possible historical population expansion. Furthermore, mismatch distributions for the northern group, southern group and the global group were unimodal and supported the hypothesis of the sudden expansion model (Figure 4). The low and nonsignificant raggedness index values (Table 5) suggested a significant fit between the observed and the expected distributions. Bayesian skyline plots for the northern group coincided exactly with the results of the global group. Bayesian skyline plots revealed late Pleistocene demographic expansion (Figure 5), which started about 160 kyr ago based on the divergence rate of 2.4%/Myr [36]. Use of the 10× faster mutation rate shortens the timing of these events by a factor of 10.

No significant heterozygotes excess (except HJ population under the IAM model) was detected under the infinite alleles model (IAM), two-phase model (TPM), and the stepwise mutation model (SMM) (Table 6). These results were consistent with the normal L-shaped distribution of allele frequency, indicating no genetic bottleneck in any of the eight populations in the recent past.

**Table 6:** Signed rank Wilcoxon test of the mutation–drift equilibrium estimated for seven microsatellite loci in populations of *A. pectinata.*

pops	ZZD	PL	LGD	RC	HD	RZ	LYG	HJ
IAM	0.53	0.71	0.41	0.47	0.53	0.66	0.77	**0.01**
TPM90	0.97	0.99	0.99	0.99	0.97	0.99	0.99	0.34
SMM	0.99	0.99	0.99	0.99	0.99	0.99	1.00	0.59

The infinite alleles model (IAM), a two-phase model (TPM90) for a 10% occurrence of multiple steps, and the stepwise mutation model (SMM).

Significant values (*P*<0.05) is highlighted in bold.

## Discussion

Genetic data can make an essential contribution to the understanding of past and current processes that have shaped the evolution and genetic structure of marine species, as well as to conservation and management strategies [57], [58]. The aim of this study was to extend knowledge on the genetic population structure and evolutionary history of *A. pectinata*, an important resource for the Chinese and Korea fishery. Patterns of genetic differentiation and population demography were estimated using both maternally-inherited COI sequences and microsatellites to reveal the components of the species population structure and population demography. We particularly focused on populations in the Yellow Sea and Bohai Sea, an area where *A. pectinata* was widely exploited and where the continental shelf were exposed during the Pleistocene ice ages and might shape the genetic variation. Indeed, we demonstrated that the COI sequences and microsatellites clarified the population demography and genetic structure of *A. pectinata*.

The most common hypotheses explaining the genetic connectivity and differentiation among different geographical populations of marine organisms are that: (i) the dispersal ability [59]–[61]; and (ii) oceanic landscapes, especially the marine currents [5], [8], [12], [62]. In the present study, high levels of genetic variability within populations and low level of genetic differentiation among populations were inferred from COI and microsatellite variation, indicating strong genetic connectivity on this spatial scale. The results of our study were generally concordant with the results of the genetic diversity and population structure analyses of *A. pectinata* along the west coast of South Korea, which show no genetic differentiation among samples probably due to the high levels of larval dispersal [21]. The results was inconsistent with that of *Scapharca broughtonii* [14], *Mactra veneriformis* [15], *Chlamys farreri* [12], and *Mactra chinensis* [8], which showed significant population genetic structure in the same region. For *Chlamys farreri* [12] and *Mactra chinensis* [8] in Bohai Sea and Yellow Sea, marine current and gyres are responsible for the significant population structure detected. Two biological characteristics of *A. pectinata* might be responsible for the high gene flow among geographical samples. First, for species with a larval phase, pelagic larval duration (PLD) is assumed to influence the scale of connectivity [63]. The longer PLD of *A. pectinata* (30 d) compared with *Chlamys farreri* (15 d) and *Mactra chinensis* (10 d) suggested that the larval dispersal potential of *A. pectinata* may be higher than that of *Chlamys farreri* and *Mactra chinensis*. Thus the larvae of *A. pectinata* might overcome barriers caused by the complex hydrology and bathymetry in Bohai Sea and Yellow Sea, which may result in high gene flow among geographical populations. Second, marine distribution patterns have been classified on a continuum from “oceanic” to “continental”, referring to requirement (or tolerance) for a suite of environmental conditions associated with primary productivity, freshwater influence and turbidity, all of which are present on continental margins and around high islands, and this has implications for the effectiveness of barriers to larvae dispersal [64]. *A. pectinata* could live in deeper ocean (to a depth of 100 m) compared to other species, so the larvae could be less impacted by ecological or hydrographical barriers to larvae dispersal, and be transported by the coastal current in longer distance than in other species.

When analyzing patterns of genetic differentiation, it is important to disentangle current gene flow from demographic processes that occurred in the population history [65], [66]. Based on COI data, a late Pleistocene population expansion of *A. pectinata* was supported by the star-like shape of minimum spanning network, the relatively clear unimodal shape of the mismatch distribution, the negative and significant Tajima’s *D* and Fu’s *F*s statistics, and the result of Bayesian skyline plots. However, if the migration rate between demes is high, population range expansion can lead to basically the same molecular signature as population demographic expansion [67], [68]. Bayesian skyline plots suggested that the population demographic expansion started about 160,000 years ago assuming a divergence rate of 2.4%/Myr for *A. pectinata*. Because mutation rate may be “time dependent” [69], and phylogenetically derived mutation rate appear to overestimate the ages of demographic events inscribed in genetic data, sometime by an order of magnitude [69]–[71]. An order of magnitude higher mutation rate than that inferred from phylogenetic data placed population expansion of *A. pectinata* at a more recent time after LGM, which was consistent with the range expansion on the East China Sea Shelf following the LGM. The late Pleistocene period (the past 1 million years) was punctuated by a series of large glacial-interglacial changes. During glacial periods, with a sea level drop of 130–150 m, the Bohai Sea and Yellow Sea were completely exposed at this time and the East China Sea was reduced to an elongated trough with an area less than a third of its present size [72], [73]. Different geographical populations of *A. pectinata* might mix together due to the geographical range contraction during the glacial period, and then expand population range when favorable conditions emerged during interglacial periods. The periodic merging of populations could enhance historical gene flow among populations. Hence both population range expansion and demographic expansion might have influenced the pattern of genetic diversity for *A. pectinata*. In this case, the large scale connectivity of *A. pectinata* could reflect the recency of this expansion and insufficient time for population divergence. Similar conclusions have been reached on some other marine invertebrates [73].

Furthermore, the low but significant genetic differentiations detected between some Chinese samples and the North Korean sample might be the result of chaotic genetic patchiness. Such chaotic genetic patchiness could be caused by any of several factors including genetic drift before recruitment and natural selection at early life stage [74]. Indeed, significant genetic differentiation are detected among age classes of *Placopecten magellanicus* [75] and between recruits from different spawning seasons in *Perna viridis* [76], which are caused by drift of larvae from different sources or large variance in reproductive success among adults.

Our results suggested that the strong connectivity and weak level of genetic structure of *A. pectinata* in Bohai Sea and Yellow Sea was probably result from both the historical recolonization (through population range expansion and demographic expansion in the late Pleistocene) and current gene flow (through the larval dispersal). Furthermore, the lack of an isolation-by-distance supported the view that the lack of significant genetic population structure was caused by high contemporary gene flow. All of the eight samples were at mutation-drift equilibrium under different mutation models indicated that current gene flow played a more important role than historical recolonization in generating current genetic structure.

## Conclusions

The results of our study revealed that, despite high level of genetic diversity within local populations of *A. pectinata* in coast of China and North Korea, there is no significant genetic differentiations among samples in Northern China and low but significant genetic differentiations between some of the Chinese samples and the North Korean sample, which might arise from both historical recolonization and current gene flow. Therefore, *A. pectinata* distributed in Northern China coast could be managed as a single unit. In addition, our results supported the conclusions of Bird *et al.* [77] about the hazards of predicting population structure and dispersal in the same marine region for species with similar life-history traits.

### Data Accessibility

A file contained the genotypes at 8 microsatellite loci of all individuals in the study is available from the Dryad Digital Repository: doi:10.5061/dryad.52hn0.

## Supporting Information

Figure S1 **Population structure of eight** ***A. pectinata*** **populations prepared using STRUCTURE program.**
(TIF)

Table S1 **Haplotype frequencies of COI gene in ten** ***A. pectinata*** **populations.**
(DOCX)

Table S2 **Matrix of pairwise** ***Φ*** **_ST_ values between ten populations based on COI datasets.**
(DOCX)
