# Supplementary figures and images for: Population Genetic Structure and Demographic History of Atrina pectinata Based on Mitochondrial DNA and Microsatellite Markers
Source: PLoS One. 2014 May 1;9(5):e95436. doi: 10.1371/journal.pone.0095436 (PMC4006771; doi:10.1371/journal.pone.0095436)

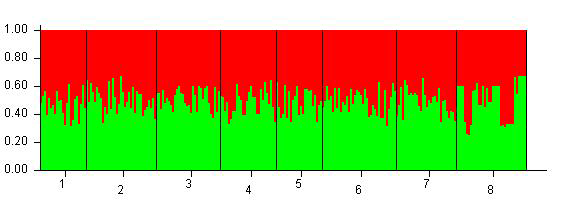

Supplement: Figure S1 — Population structure of eight A. pectinata populations prepared using STRUCTURE program. (TIF) [file pone.0095436.s001.tif]
