# Supplementary material for: Population Genetic Structure and Demographic History of Atrina pectinata Based on Mitochondrial DNA and Microsatellite Markers
Source: PLoS One. 2014 May 1;9(5):e95436. doi: 10.1371/journal.pone.0095436 (PMC4006771; doi:10.1371/journal.pone.0095436)
Supplement: Table S1 — Haplotype frequencies of COI gene in ten A. pectinata populations. (DOCX) [file pone.0095436.s002.docx]

**Table S1 The haplotype frequencies of mtCOI gene in ten *A. pectinata* populations**

|  | GenBank accession number | ZZD | PL | LGD | RC | HD | RZ | LYG | HJ | ZS | FZ | Total |
| --- | --- | --- | --- | --- | --- | --- | --- | --- | --- | --- | --- | --- |
| Hap1 | KJ462138 |  |  |  |  |  |  |  | 1 |  |  | 1 |
| Hap2 | KJ462139 | 1 |  |  |  |  |  |  | 3 |  |  | 4 |
| Hap3 | KJ462140 |  |  |  | 1 |  |  |  | 2 |  |  | 3 |
| Hap4 | KJ462141 |  |  |  |  |  |  |  | 3 |  |  | 3 |
| Hap5 | KJ462142 | 7 | 12 | 11 | 9 | 5 | 5 | 9 | 1 | 6 | 8 | 73 |
| Hap6 | KJ462143 |  |  |  |  |  |  |  | 4 |  |  | 4 |
| Hap7 | KJ462144 |  | 1 |  |  |  | 1 |  | 3 |  |  | 5 |
| Hap8 | KJ462145 |  |  |  |  |  |  |  | 2 |  |  | 2 |
| Hap9 | KJ462146 |  |  |  |  |  |  |  | 1 |  |  | 1 |
| Hap10 | KJ462147 |  |  |  |  | 1 |  |  | 2 |  |  | 3 |
| Hap11 | KJ462148 |  |  |  |  | 1 | 2 |  | 1 |  | 1 | 5 |
| Hap12 | KJ462149 | 3 |  | 2 | 2 | 1 | 2 | 1 | 1 | 2 | 2 | 16 |
| Hap13 | KJ462150 |  |  |  |  |  |  | 1 |  |  |  | 1 |
| Hap14 | KJ462151 |  |  |  |  |  |  | 1 |  |  |  | 1 |
| Hap15 | KJ462152 |  |  |  |  |  |  | 1 |  |  |  | 1 |
| Hap16 | KJ462153 |  | 1 |  | 1 | 1 |  | 2 |  |  | 1 | 6 |
| Hap17 | KJ462154 | 1 |  |  |  | 1 | 1 | 1 |  |  |  | 4 |
| Hap18 | KJ462155 |  |  |  |  | 1 |  | 1 |  |  |  | 2 |
| Hap19 | KJ462156 |  |  |  |  |  |  | 1 |  |  |  | 1 |
| Hap20 | KJ462157 |  |  |  |  |  |  | 1 |  |  |  | 1 |
| Hap21 | KJ462158 |  |  |  |  |  |  | 1 |  |  |  | 1 |
| Hap22 | KJ462159 |  |  |  |  |  | 2 |  |  |  | 1 | 3 |
| Hap23 | KJ462160 |  |  |  |  | 1 | 1 |  |  |  |  | 2 |
| Hap24 | KJ462161 |  |  |  |  |  | 2 |  |  |  |  | 2 |
| Hap25 | KJ462162 |  |  |  |  |  | 1 |  |  |  |  | 1 |
| Hap26 | KJ462163 |  |  |  |  | 1 | 1 |  |  |  |  | 2 |
| Hap27 | KJ462164 |  |  |  |  |  | 1 |  |  |  |  | 1 |
| Hap28 | KJ462165 |  |  |  |  |  | 1 |  |  |  |  | 1 |
| Hap29 | KJ462166 |  |  |  |  |  | 1 |  |  |  |  | 1 |
| Hap30 | KJ462167 |  |  |  |  | 1 |  |  |  |  |  | 1 |
| Hap31 | KJ462168 |  |  |  |  | 1 |  |  |  | 1 |  | 2 |
| Hap32 | KJ462169 |  |  |  | 1 | 1 |  |  |  |  |  | 2 |
| Hap33 | KJ462170 |  |  |  |  | 1 |  |  |  |  |  | 1 |
| Hap34 | KJ462171 |  |  |  |  | 1 |  |  |  |  |  | 1 |
| Hap35 | KJ462172 |  |  |  |  | 1 |  |  |  |  |  | 1 |
| Hap36 | KJ462173 |  |  |  |  | 1 |  |  |  |  |  | 1 |
| Hap37 | KJ462174 |  |  |  | 1 |  |  |  |  |  |  | 1 |
| Hap38 | KJ462175 |  |  | 1 | 1 |  |  |  |  |  |  | 2 |
| Hap39 | KJ462176 |  |  |  | 1 |  |  |  |  | 1 |  | 2 |
| Hap40 | KJ462177 |  |  |  | 1 |  |  |  |  |  |  | 1 |
| Hap41 | KJ462178 | 1 |  |  | 1 |  |  |  |  |  |  | 2 |
| Hap42 | KJ462179 |  |  |  | 1 |  |  |  |  |  |  | 1 |
| Hap43 | KJ462180 | 1 |  | 2 |  |  |  |  |  |  |  | 3 |
| Hap44 | KJ462181 |  |  | 1 |  |  |  |  |  |  |  | 1 |
| Hap45 | KJ462182 |  |  | 1 |  |  |  |  |  |  |  | 1 |
| Hap46 | KJ462183 |  |  | 1 |  |  |  |  |  |  |  | 1 |
| Hap47 | KJ462184 |  | 1 | 1 |  |  |  |  |  |  |  | 2 |
| Hap48 | KJ462185 |  |  | 1 |  |  |  |  |  |  |  | 1 |
| Hap49 | KJ462186 |  | 1 |  |  |  |  |  |  |  |  | 1 |
| Hap50 | KJ462187 |  | 1 |  |  |  |  |  |  |  |  | 1 |
| Hap51 | KJ462188 |  | 1 |  |  |  |  |  |  |  |  | 1 |
| Hap52 | KJ462189 |  | 1 |  |  |  |  |  |  |  |  | 1 |
| Hap53 | KJ462190 |  | 1 |  |  |  |  |  |  |  |  | 1 |
| Hap54 | KJ462191 | 1 |  |  |  |  |  |  |  | 1 |  | 2 |
| Hap55 | KJ462192 | 1 |  |  |  |  |  |  |  |  |  | 1 |
| Hap56 | KJ462193 | 1 |  |  |  |  |  |  |  |  |  | 1 |
| Hap57 | KJ462194 | 1 |  |  |  |  |  |  |  |  |  | 1 |
| Hap58 | KJ462195 | 1 |  |  |  |  |  |  |  |  |  | 1 |
| Hap59 | KJ462196 | 1 |  |  |  |  |  |  |  |  | 1 | 2 |
| Hap60 | HQ449258 |  |  |  |  |  |  |  |  |  | 1 | 1 |
| Hap61 | HQ449261 |  |  |  |  |  |  |  |  |  | 1 | 1 |
| Hap62 | HQ449310 |  |  |  |  |  |  |  |  |  | 1 | 1 |
| Hap63 | HQ449311 |  |  |  |  |  |  |  |  |  | 1 | 1 |
| Hap64 | HQ449312 |  |  |  |  |  |  |  |  |  | 1 | 1 |
| Hap65 | HQ449313 |  |  |  |  |  |  |  |  |  | 1 | 1 |
| Hap66 | HQ449314 |  |  |  |  |  |  |  |  |  | 1 | 1 |
| Hap67 | HQ449315 |  |  |  |  |  |  |  |  |  | 1 | 1 |
| Hap68 | HQ449316 |  |  |  |  |  |  |  |  |  | 1 | 1 |
| Hap69 | HQ449317 |  |  |  |  |  |  |  |  |  | 1 | 1 |
| Hap70 | HQ449322 |  |  |  |  |  |  |  |  |  | 1 | 1 |
| Hap71 | HQ449285 |  |  |  |  |  |  |  |  | 1 |  | 1 |
| Hap72 | HQ449294 |  |  |  |  |  |  |  |  | 1 |  | 1 |
| Hap73 | HQ449295 |  |  |  |  |  |  |  |  | 1 |  | 1 |
| Hap74 | HQ449296 |  |  |  |  |  |  |  |  | 1 |  | 1 |
| Hap75 | HQ449298 |  |  |  |  |  |  |  |  | 1 |  | 1 |
| Hap76 | HQ449300 |  |  |  |  |  |  |  |  | 1 |  | 1 |
| Hap77 | HQ449301 |  |  |  |  |  |  |  |  | 1 |  | 1 |
| Hap78 | HQ449302 |  |  |  |  |  |  |  |  | 1 |  | 1 |
| Hap79 | HQ449303 |  |  |  |  |  |  |  |  | 1 |  | 1 |
| Total | - | 20 | 20 | 21 | 20 | 20 | 21 | 20 | 24 | 20 | 25 | 211 |
