# Supplementary material for: Population Genetic Structure and Demographic History of Atrina pectinata Based on Mitochondrial DNA and Microsatellite Markers
Source: PLoS One. 2014 May 1;9(5):e95436. doi: 10.1371/journal.pone.0095436 (PMC4006771; doi:10.1371/journal.pone.0095436)
Supplement: Table S2 — Matrix of pairwise Φ ST values between ten populations based on COI datasets. (DOCX) [file pone.0095436.s003.docx]

|  | ZZD | PL | LGD | RC | HD | RZ | LYG | HJ | ZS | FZ |
| --- | --- | --- | --- | --- | --- | --- | --- | --- | --- | --- |
| ZZD | - |  |  |  |  |  |  |  |  |  |
| PL | -0.0174 | - |  |  |  |  |  |  |  |  |
| LGD | 0.0015 | 0.0177 | - |  |  |  |  |  |  |  |
| RC | -0.0188 | 0.0023 | -0.0065 | - |  |  |  |  |  |  |
| HD | -0.0050 | 0.0044 | 0.0025 | -0.0186 | - |  |  |  |  |  |
| RZ | -0.0220 | 0.0439 | 0.0059 | -0.0017 | -0.0177 | - |  |  |  |  |
| LYG | 0.0004 | 0.0165 | 0.0215 | -0.0289 | -0.0188 | 0.0138 | - |  |  |  |
| HJ | 0.0519 | **0.0745** | 0.0412 | 0.0145 | 0.0081 | -0.0019 | 0.0242 | - |  |  |
| ZS | -0.0225 | -0.0065 | -0.0094 | -0.0177 | -0.0165 | -0.0125 | 0.0012 | 0.0202 | - |  |
| FZ | -0.0155 | -0.0197 | 0.0313 | 0.0047 | 0.0008 | 0.0405 | 0.0140 | **0.0805** | -0.0040 | - |

**Table S2 Matrix of pairwise *Φ*_ST_ values between ten populations based on mtCOI datasets**

Significant values after Benjamini–Yekutieli correction based on the false discovery rate approach (P < 0.0120) are highlighted in bold.
